# Xanthogranulomatous inflammation involving latissimus dorsi donor site and implant breast reconstruction: case report and literature review

**DOI:** 10.1186/1477-7819-10-166

**Published:** 2012-08-20

**Authors:** Tasadooq Hussain, Bilal Elahi, Ervine Long, Tapan Mahapatra, Penelope L McManus, Peter J Kneeshaw

**Affiliations:** 1 Clinical Research Fellow Breast Surgery, Cancer Biology Proteomic Groups, University of Hull, HYMS, Hull, UK; 2 Breast Unit, Castle Hill Hospital, Hull and East Yorkshire NHS Trust, Hull, UK

## Abstract

Xanthogranulomatous inflammation is a rare clinico-pathological condition involving many organ systems. Breast involvement with this rare condition reported from a few cases of mastitis has been limited to only microscopic involvement on histology. We would like to report an unusual presentation of this inflammatory process presenting as a solid lump mimicking malignancy in latissimus dorsi donor site scar and implant-based breast reconstruction as a result of a ruptured silicone gel implant. To our knowledge there have been no previous reports on similar presentation published in the literature. This case highlights a rare complication of a leaked silicone gel implant triggering a xanthomatous response in the absence of the usual infective or obstructing etiologies. This condition is of benign nature with complete clearance on surgical excision and excellent clinical prognosis reported from other organ involvement.

## Background

Xanthogranulomatous inflammation involves various organ systems such as the gall bladder, kidney, pancreas, appendix, eyes, female genital tracts, colon, and urachus [1-8]; breast involvement seen with this inflammation has been limited to only few rare cases of mastitis. We would like to report an unusual involvement of this inflammatory process presenting as a solid lump resembling a malignancy in latissimus dorsi donor site scar and breast implant in a previously treated breast cancer patient. Occurrence of this inflammation from a ruptured implant and silicone gel leak in not known; to our knowledge, there have been no previous reports on similar presentations.

## Case presentation

**Figure 1:**
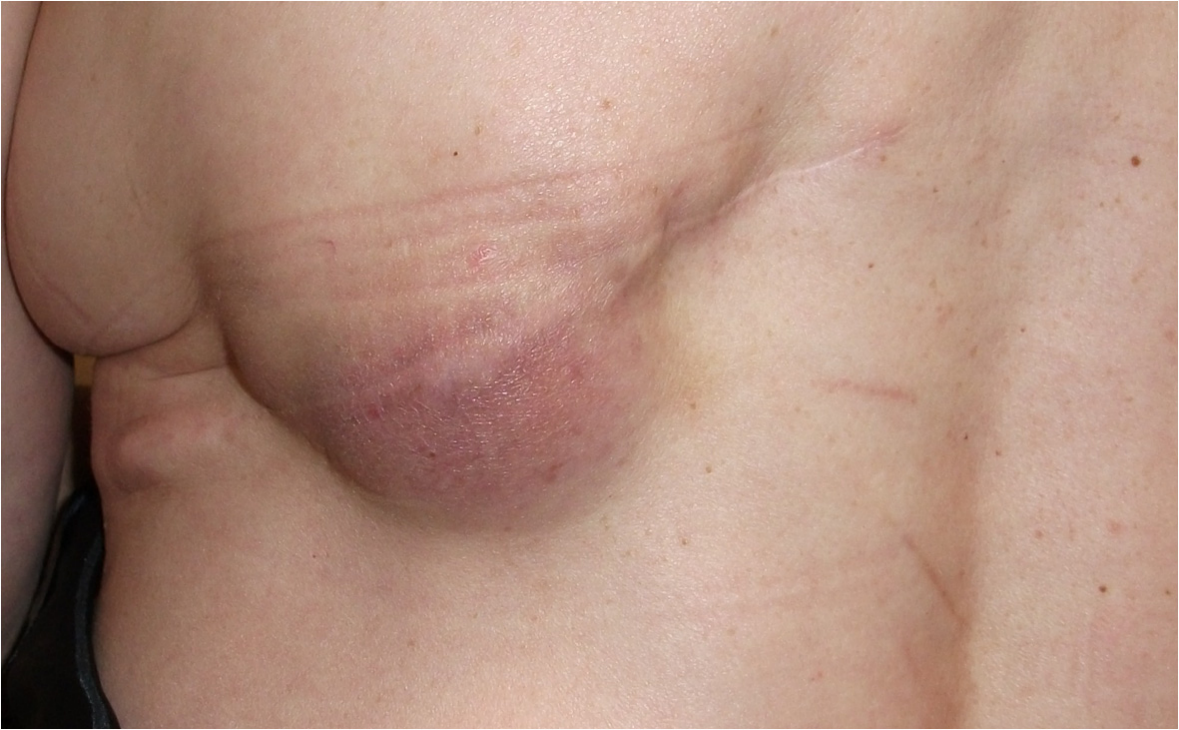
Palpable abnormal lump arising in the latissimus dorsi (LD) donor site scar.

A 56-year-old lady presented with an abnormal mass arising from her latissimus dorsi (LD) donor site scar (Figure 1) and hardening of the left reconstructed breast. She had no co-morbidities apart from anxiety disorder and breast cancer treated 10 years ago. She had a left mastectomy and immediate implant-assisted LD reconstruction. The tumor was grade 3 estrogen receptor positive. The adjuvant treatment was chemotherapy and an aromatase inhibitor (anastrazole). Her routine follow-up was uneventful and she had been discharged.

**Figure 2:**
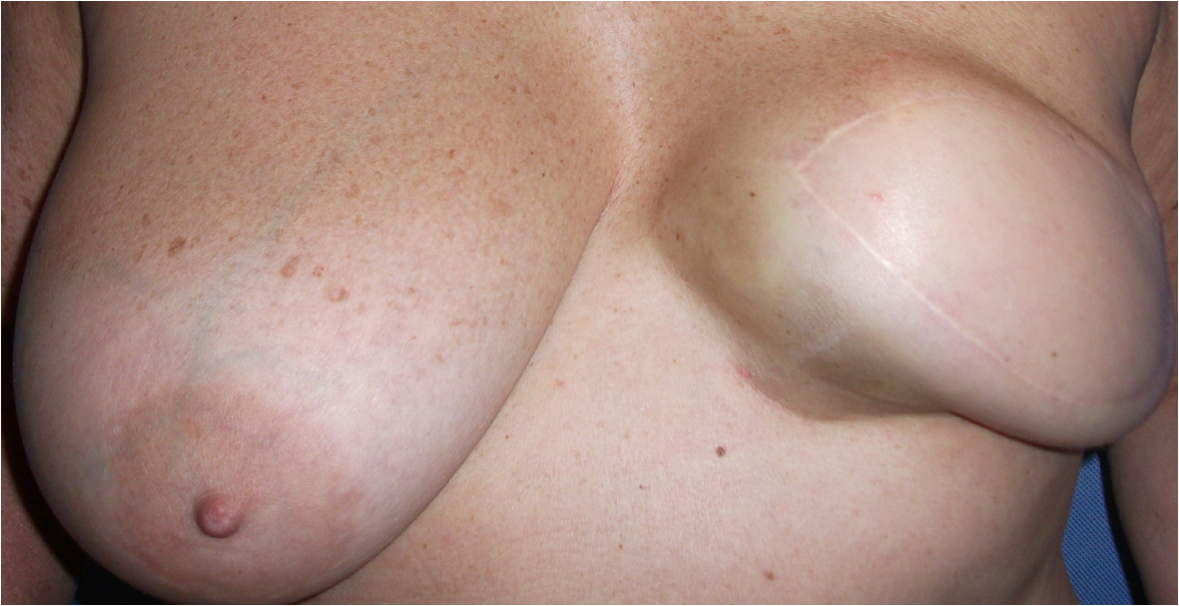
Abnormal looking left breast reconstruction with marked capsular contracture.

On examination she had a 10 cm mass arising from the donor site scar. The mass was well defined with an area of softness on the anterior surface. The left reconstructed breast had a marked capsular contracture (Figure 2) and a superficially placed implant under the thinned medial aspect of the LD flap. The donor site scar changes were considered highly suspicious of cancer recurrence or a new primary with no clinical evidence of any recurrent disease in the left reconstructed breast and axilla.

**Figure 3:**
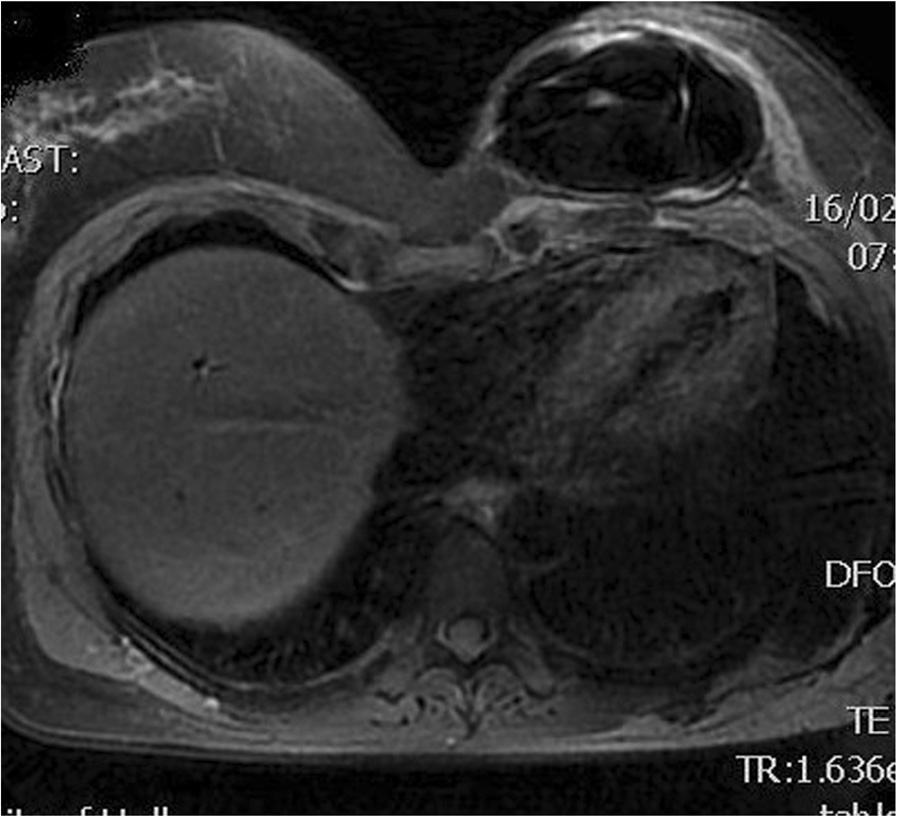
**Magnetic resonance imaging** (**MRI) showing capsular contracture of left breast implant and posterior latissimus dorsi (LD) mass.**

Initial investigation of the mass with ultrasound (US) showed solid tissue within the mass with a central fluid collection that on fine needle aspiration showed necrotic debris. US findings were confirmed by the contrast-enhanced computer tomography (CT) scan with no evidence of any distant metastasis. A magnetic resonance imaging (MRI) scan (Figure 3) of the breast and the lump showed features of implant encapsulation and nodularity, with areas of high-contrast uptake in the breast reconstruction and LD scar suggesting breast cancer recurrence. The core biopsy of the back mass lesion showed features of granular histiocytes mixed with multinucleated giant cells and abundant cholesterol clefts. These changes reflected a florid reactive inflammatory process of xanthomatous nature with no evidence of any malignancy.

**Figure 4:**
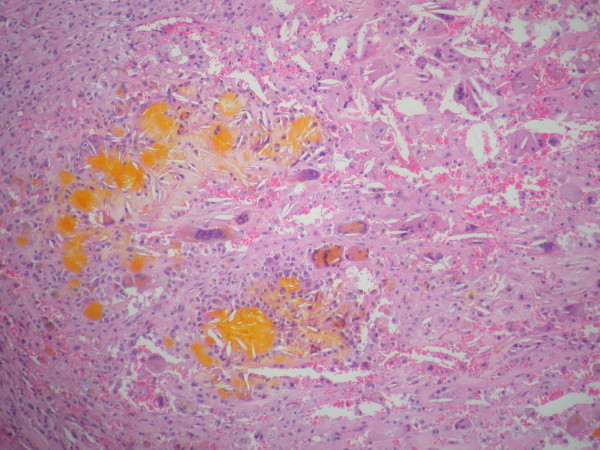
**Photomicrograph of a hematoxylin and eosin slide showing the characteristic appearances of xanthogranulomatous inflammation.** Sheets of histiocytes are interspersed with multinucleate giant cells, empty cholesterol clefts and yellow hematoidin pigment (original magnification × 40).

**Figure 5:**
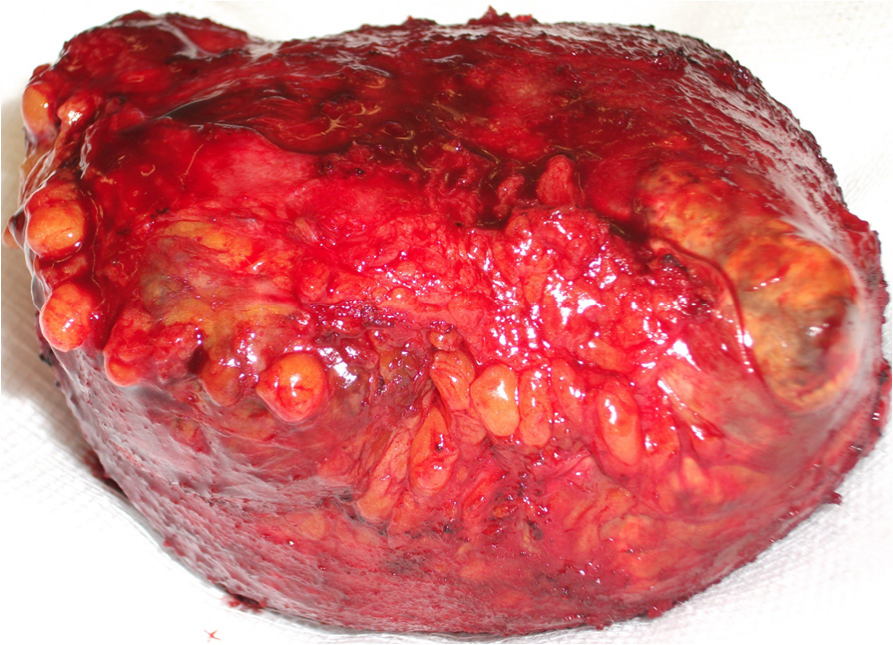
Operative specimen showing capsular xanthomas.

Excision biopsy of the back mass lesion and revision of the reconstructed breast for definitive histological diagnosis was performed in two stages. In stage one, the mass was excised with a transverse ellipse and the wound closed with advancement flaps. The microscopic appearances of the excised lesion were similar to core biopsy findings with xanthoma-laden large macrophages interlaced with inflammatory cells and no malignancy (Figure 4). Findings of this nature on histology were pointing towards a chronic inflammatory etiology secondary to possibly silicone leak from the breast implant. The breast reconstruction was revised in the second stage; operative findings confirmed rupture in the implant with posterior tracking of silicone and features of old hemorrhage. The leaking implant was removed and capsulectomy performed. The LD flap was medially advanced and the breast reconstruction was completed by using a style 150 expandable implant prosthesis. Intra-operative characteristic yellow discoloration of both the implant and capsule (Figure 5) indicated involvement with strong xanthomatous inflammation. These findings were confirmed later on histology with no evidence of malignancy noted in the excised breast tissue. The patient recovered well from both the procedures and required no further treatment for the condition after final histology.

Xanthogranulomatous inflammation is a benign pathological condition considered to be a primary disease process of unknown etiology. Various organ system can be involved to varying degrees; most commonly affected are the kidneys and gall bladder with a few rare cases of colonic involvement. Evidence of breast involvement comes from cases of inflammatory mastitis [9] showing features of xanthomatous change on microscopy. There is no associated age- or sex-related preponderance with the condition, with a few cases from an early pediatric age group also noted [10].

The exact etiology of the disease is unknown; organ obstruction, suppurative infections and hemorrhage trigger tissue damage within the involved organs, usually eliciting a microscopic response of the disease process. The case study by Luc and colleagues [11] following long-standing infections with mycoplasma hominis supports the infective etiology theory of this condition. These triggers, either on their own or synchronously (as postulated in the obstructive hypothesis of gall bladder and kidney pathologies), cause extensive chronic inflammation of the involved organ resulting in a xanthogranulomatous transformation. A similar obstructive trigger would also explain the changes in xanthogranulomatous appendicitis following chronic inflammation of the obstructed lumen with faecolith.

Xanthogranulomatous inflammation disease presentation is variable, with both localized and diffuse responses. A diffuse inflammation is the more severe form of the disease, resulting in a complete obliteration of the native parenchymal tissue and marked fibrosis which clinically presents as solid lumps. Xanthogranulomatous inflammationcan also have a cutaneous involvement which can be primary or secondary [12]. Secondary disease represents a severe form of an internal disease process and, clinically, patients present with a discharging skin lesion that communicates through an underlying fistula with the involved organ.

Radiological diagnosis of xanthogranulomatous inflammation using US and CT is challenging. Diagnostic accuracy can be increased using contrast-based imaging, as demonstrated using sonazoid contrast enhancing agent with US scan in the diagnosis of chronic inflamed and thickened gall bladders, and CT urography studies for renal xanthogranulomatous inflammatory disease [13,14]. The characteristic findings on imaging are the areas of low attenuation nodules corresponding to the xanthomatous change on histology.

The microscopic appearances of xanthogranulomatous inflammation on histology show the characteristic multinucleate giant cells interspersed with lipid-laden macrophages which impart the characteristic yellow macroscopic appearance. This appearance, however, should be differentiated from a pseudo xanthomatous inflammation or malakoplakia characterized by Michaelis-Gutman bodies which stain positive with Von Kossa calcium and Prussian blue stains [15].

In our case, an implant leak triggered the inflammatory process. Presentation of a well localized mass in the LD back scar was a result of an extensive tissue inflammation and fibrosis from posterior tracking of the leaked silicone. However, what surprisingly was absent in our case was the postulated triggers of obstruction or infection at surgery; gram staining of both implant and back tissue was negative too. The pre-operative imaging was mostly helpful in delineating the anatomy of the mass and excluding any systemic spread from previously treated breast primary; failure to accurately diagnose the condition confirms the limitations of both CT and MRI in these cases.

The recommended treatment of solid lumps with diffuse involvement is usually surgical as this condition is not pre-malignant [16]. A complete surgical excision negates the need for any further treatment. There is no evidence in the literature to support any recurrence in the native or other organs following the primary surgical treatment.

## Conclusions

The silicone gel-filled breast implants are safe with a prolonged life span; however, implant-related problems increases with time (usual complications are capsular contractures, asymmetry and ruptures) and, therefore, surgeons should be wary of these unusual presentations when dealing with the complications of long-standing implants.

## Consent

Written informed consent was obtained from the patient for publication of this case report and accompanying images. A copy of the written consent is available for review by the Editor-in-Chief of this journal.

## Abbreviations

CT, computer tomography; LD, latissimus dorsi; MRI, magnetic resonance imaging; US, ultrasound..

## Competing interests

The authors report that they have no conflict of interests.

## Authors’ contributions

TH did the literature search and has been a major contributor in writing the manuscript. PJK did the final draft proof reading and approval. EL provided the pathology images. All authors read and approved the final manuscript.
